# Evaluation of a Childhood Obesity Program Serving a High-Need Population in Brooklyn, New York Using Survival Analysis

**DOI:** 10.3390/ijerph20095723

**Published:** 2023-05-04

**Authors:** Alecia James, Aimee Afable, Nagla Bayoumi, Sarita Dhuper

**Affiliations:** 1 Department of Epidemiology and Biostatistics, School of Public Health, SUNY Downstate Health Sciences University, 450 Clarkson Ave, Brooklyn, NY 11203, USA; 2 Department of Community Health Sciences, School of Public Health, SUNY Downstate Health Sciences University, 450 Clarkson Ave, Brooklyn, NY 11203, USA; 3 Department of Pediatrics, SUNY Downstate Health Sciences University, 450 Clarkson Ave, Brooklyn, NY 11203, USA

**Keywords:** obesity, childhood obesity, obesity intervention, survival analysis

## Abstract

In this study, we used survival analysis to evaluate whether contact hours intensity was associated with a reduction in time to improvement of various BMI metrics over a 5-year follow-up period at the Live Light Live Right pediatric obesity program in Brooklyn, New York. This was a single-center retrospective longitudinal study of 406 patients during 2010–2016. Participants were categorized based on hours of exposure to Live Light Live Right’s interventions; high contact hours (≥50 h) vs. low contact hours (<50 h). At baseline, 88% of patients in the high contact hour group had severe obesity and the mean age for this group was 10.0 ± 2.66. High contact hours were independently associated with a shorter time to BMI improvement in the sample. There was also a significant association between high contact hours and a longer duration in the improved state. Survival analysis was successful in evaluating the efficacy of the Live Light Live Right Program and demonstrated a positive association between greater intervention intensity and a healthier metabolic profile. Patients’ active engagement in a robust treatment model exemplified by Live Light Live Right is recommended to address the childhood obesity crisis in central Brooklyn.

## 1. Introduction

The obesity epidemic in the U.S. is a major public health concern; studies have reported an obesity prevalence of 19.3% in youths, which includes 6.1% with severe obesity [1]. Reports on severe obesity in New York City youth have indicated a disproportionate burden of disease among children of color [2]. Central Brooklyn is an area of concern, as it is comprised of predominantly low-income African American and Afro-Caribbean neighborhoods. It is also one of three New York City Department of Health districts identified as high-need with a disproportionate burden of chronic disease. The obesity rates in kindergarten through 8th grade (K—8) children in the central Brooklyn areas of Brownsville and East New York—some of the most underserved neighborhoods in New York City—of 23% and 25%, respectively, exceed those of the overall Brooklyn and New York City [3,4].

The American Heart Association (AHA) has emphasized that in comparison to youths with low obesity, children with severe obesity have a more adverse cardiovascular risk factor profile and show early signs of vascular dysfunction and subclinical atherosclerosis [5]. Furthermore, studies suggest that severe obesity in childhood may increase the likelihood of obesity in adulthood [6]. Significantly, the lifetime cost of obesity remains a huge burden: obesity-related medical spending in the U.S. in 2014 was an estimated USD 149 billion [7]. Thus, the need for targeted childhood obesity interventions in central Brooklyn becomes evident, if we are to address this epidemic, its associated adverse health outcomes, and economic burden.

It is well-documented that intensive lifestyle modification interventions compared to standard of care lead to a significant and clinically meaningful reduction in weight in children and adolescents [8,9,10,11,12]. Further lifestyle modification in high-risk adults has been shown to delay the onset of obesity-related conditions, including diabetes and cardiovascular risk factors [13,14,15].

International recommendations agree that the core elements of any initiative to address pediatric obesity should involve the whole family and include nutrition education, behavior modification, and promotion of physical activity [8,9,10,11,12]. Of note, the United States Preventive Task Force (USPTF) recommends obesity screening and behavioral interventions in children and adults to promote improvement in weight status [16,17]. The transcreation framework for addressing health disparity in populations recommends engaging community partners in intervention delivery, making these interventions more sustainable, appealing, and culturally sensitive [18]. Additionally, the National Academy of Medicine (NAM) has proposed a framework that integrates clinical and community systems to prevent and manage obesity [19].

Live Light Live Right (LLLR) is an evidence-based childhood obesity program—serving the central Brooklyn area—that takes a sustainable approach to obesity management. It is the only tertiary-care childhood obesity program in Brooklyn, New York, and 85% of the patients seen have severe obesity. Thus, under the transcreation framework [18], it is essential to be able to perform a “real world” assessment of the effectiveness of this intervention program, which could serve as a model for other disproportionately affected areas. Traditionally, pretest-posttest designs have been used to evaluate the effectiveness of disease management intervention programs. However, this approach has been deemed relatively weak—it is prone to loss to attrition and the effect of confounding factors [20]. Similarly, in conventional cohort analyses, loss-to-follow-up reduces the analytical sample and introduces bias [21].

To address some of these shortcomings, this study utilized the method of survival analysis, a robust statistical approach, to evaluate the effectiveness of the LLLR childhood obesity program. Unlike conventional retrospective cohort studies which often suffer from loss-to-follow-up and selection bias, survival analysis permits analysis of loss-to-follow-up by accounting for the time subjects are in the study. In survival analysis, the outcome of interest is the “time to event [22,23],”and using time to event as an outcome can provide more clinical information in lieu of the binary indicator of whether an event occurs [23]. One can assess the efficacy of an intervention based on how quickly it takes to achieve the event, for example. In this study, we use survival analysis to assess the time taken to improve BMI_z_, BMI%_95,_ and ΔBMI metrics for 12 consecutive months based on contact hours received at LLLR.

## 2. Materials and Methods

### 2.1. Intervention Methods

#### LLLR Individualized Treatment Model

**Figure 1:**
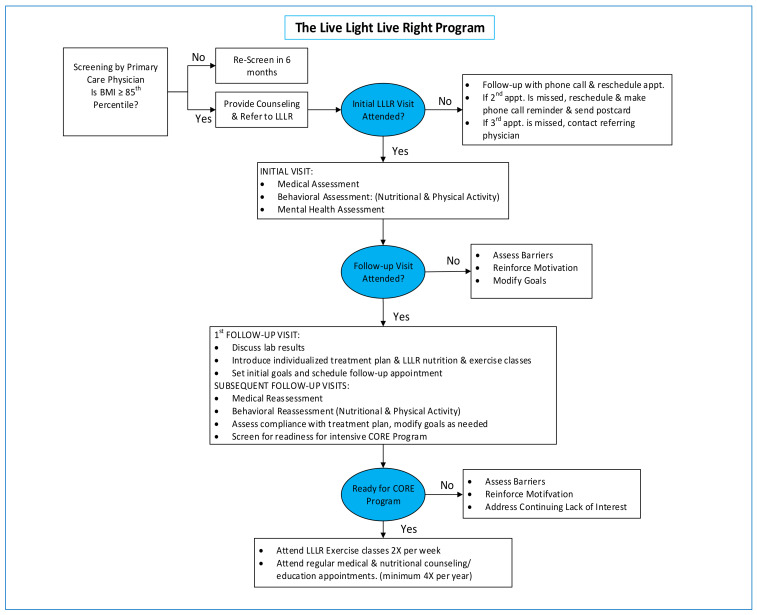
The Live Light Live Right Treatment Model.

Figure 1 illustrates the schematic of the LLLR treatment program. Children between the ages of 2 and 19 with a body mass index (BMI) ≥85th percentile for age and sex can be referred to the program. LLLR provides individualized obesity treatment plans with three core elements: (1) medical evaluation and treatment of patients at its multi-disciplinary obesity specialty clinic; (2) family nutritional and behavioral counseling and age-appropriate wellness education; and (3) referral to supervised exercise programs at a number of local gyms and community-based organizations. Behavioral treatment of obesity at LLLR incorporates strategies such as goal setting, self-monitoring, stimulus control, and problem-solving [24]. A detailed overview of LLLR’s intervention method can be found in the literature [21].

### 2.2. Study Methods

#### Study Design

This was a single-center retrospective longitudinal study; data for this study were obtained from the electronic medical records of patients in the LLLR program. The Institutional Review Board at SUNY Downstate Medical Center approved this study as secondary research which waived the requirement for informed consent.

### 2.3. Subjects

A total of 406 participants who were enrolled in the LLLR program for a minimum of 1 year between 2010 and 2016 were eligible for the study. Participants not enrolled during this period for a minimum of one year were excluded from the study.

#### 2.3.1. Contact Hour Grouping (Main Predictor)

Contact hours are determined based on clinical visits and participation in LLLR’s exercise program, core components of their individualized treatment model. The initial clinical visit counts for 3 contact hours, and subsequent visits equated to 2 contact hours. Each exercise session accounts for 1 contact hour. This division by contact hours categorized participants into a high contact hour group comprising those receiving 50 or more hours of clinical visits and exercise (150 patients), and a low contact hour group with those receiving less than 50 h of the intervention (256 patients) throughout a 5-year follow-up period. The United States Preventive Task Force (USPTF) found that comprehensive behavioral modifications of a minimum of 26 contact hours over a 2–12-month period result in improvement in weight status [16]. They further noted that “behavioral interventions with 52 contact hours or more demonstrated greater weight loss and improvements in metabolic and cardiovascular risk factors [16]”. The current research used 50 contact hours as a cut-point as an option to round the 52 contact hours. Based on experience, we do not feel that 26 contact hours were enough to show an impact. Furthermore, since the majority of the clinic’s patient population had severe obesity, it was important that they received an intervention that provided an adequate amount of exposure. Additionally, since this is a long-term study with a 5-year follow-up period, 50 contact hours would be more ideal for the high contact hour group than a threshold of 26 contact hours.

#### 2.3.2. Event Endpoint (Primary Outcome)

This was defined as the first recorded time of improving the level of BMI_z_, BMI%_95_, and ΔBMI metrics/risk factors below the recorded baseline level and staying in that improved state for at least 12 months. The maximum follow-up time for this study was 60 months after the initial visit.

Secondary Outcomes: Improvement in systolic and diastolic hypertension for 12 consecutive months.

This was defined as the first recorded time of improving systolic and diastolic hypertension (decreasing systolic and diastolic blood pressure) from the baseline measurements of these metrics that is sustained for 12 months.

#### 2.3.3. Censoring

Participants who did not experience the “event” have been right-censored at the time of the last visit from the initial visit, before the 60-month period (loss-to-follow-up censoring). Additionally, participants who made it to the end of the study and did not experience the event were right-censored (end of study censoring).

#### 2.3.4. Measurement of Outcomes

BMI was determined based on patients’ recorded height and weight. It was calculated using the formula: weight (kg)/height (m^2^). From this, we determined the BMI percentile, which is a child’s BMI relative to that of US children of the same sex and age. [25]. BMI %_95_ is a child’s BMI percentile relative to the 95th percentile of BMI of US children of the same sex and age. This was calculated using the CDC’s growth charts.

BMI percentiles were used to determine weight statuses. Healthy weight was defined as BMI ranging from the 5th percentile to less than the 85th percentile; overweight: 85th percentile ≤ BMI < 95th percentile; obesity: 95th percentile ≤ BMI < 120% of the 95th percentile; and severe obesity: BMI ≥ 120% of the 95th percentile.

BMI_z_ scores were another BMI indicator used. These are standard deviation scores that may be used to quantify how far BMI values are from population means based on sex and age [26]. BMI_z_ was calculated using the LMS method [26].

The third BMI metric used was ΔBMI. Delta BMI was calculated from patients’ BMI and BMI%_95_ by subtracting BMI%_95_ from the BMI values. Since a child’s BMI%_95_ value is based on age (and sex), it means that this metric can change with a child’s age, even if their BMI stays constant. Thus, delta BMI is a metric that captures the differences in BMI and BMI%_95_ over time.

BMI _z_ is the most frequently used of these BMI metrics in children, however, it has drawbacks as it results in the mapping of a wide range of very high BMI values to similar z-scores [26,27]. Additionally, there is a limit to the maximum z score that can be obtained at any sex or age [26,27].

The current research used three different BMI metrics to allow for comparisons with other studies, as researchers vary with their choice of BMI metric.

A detailed overview of how the anthropometric measures and health indicators were obtained (and defined) at LLLR can be found in the literature [21].

Additional anthropometric measures and health indicators taken were waist circumference, systolic and diastolic hypertension, lipid levels, total cholesterol, high- and low-density lipoprotein, triglycerides, and fasting glucose.

Reference values from the National Cholesterol Education Program’s Pediatric Panel report were used to determine abnormalities for waist circumference, blood pressure, and lipid levels, as articulated in our prior research [21].

Hypertension was indicated by systolic blood pressure (SBP) or diastolic blood pressure (DBP) ≥ the 90th percentile for age, sex, and height. A waist circumference ≥ 90th percentile for age and sex was defined as abnormal.

Abnormal lipid levels were indicated by: total cholesterol ≥ 160 mg/dL; high-density lipoprotein (HDL) levels ≤ 40 mg/dL; low-density lipoprotein (LDL) levels ≥ 110 mg/dL; and total triglycerides ≥ 110 mg/dL for children aged 12 or older or ≥ 90th percentile for age and sex. An elevated glucose level of ≥110 mg/dL was defined as abnormal. The presence of three or more components of the metabolic syndrome indicated an elevated metabolic risk. The components included: an abnormal waist circumference; systolic hypertension or diastolic hypertension; an abnormally low HDL level; abnormal triglycerides; and elevated fasting glucose levels.

All anthropometric measures and health indicators were collected at baseline, and at the medical reassessment follow-up visit after at least 12 consecutive months of program participation.

#### 2.3.5. Measurement of Covariates

The main covariates are age, sex, and race/ethnicity, and they were self-reported at the initial visit.

### 2.4. Statistical Analysis

Continuous variables are expressed as means and standard deviations, and categorical variables are expressed as frequencies and percentages. Independent samples *t*-tests were used for comparing continuous variables, and chi-square tests were used to compare differences in the categorical variables.

We also used independent samples *t*-tests to compare the average times of staying in the improved state for various risk factors, based on contact hour exposure as this information can be informative in chronic disease management.

### 2.5. Survival Analysis

#### 2.5.1. Bivariate Analysis

The Kaplan-Meir method was used to estimate the survival function for subgroups by select risk factors. The survival curves for the low and high contact hour groups are based on the records of 406 patients as noted earlier. The log-rank test was used to determine if the curves differed significantly.

#### 2.5.2. Multivariate Analysis

To control for the effect of confounders such as age and sex, we used a Cox Proportional Hazards model; the Breslow method was used to estimate the hazard function. Three separate models were created. The risk factor/BMI metric assessed in the first model was BMI%_95_. The main predictor was contact hours, a dichotomous variable, with low and high contact hours as the two levels. The covariates used were age, a continuous variable, and sex, coded as male or female. The outcome assessed was “time to event.” The second and third models were identical to the first, with the exception of BMI_z_ score and ΔBMI as the BMI metric/risk factors in these models, respectively. For all analyses, a two-sided *p*-value of <0.05 was considered statistically significant. All analyses were performed using IBM SPSS software version 27.

## 3. Results

### 3.1. Description of Sample

Table 1 shows the baseline characteristics of the 406 patients assessed by contact hours exposure. Of these participants, those receiving high contact hours were younger, with a mean age of 10, compared to a mean age of 11 in the group receiving low contact hours. This difference was statistically significant (*p* < 0.001). Males accounted for 43% of the sample and 57% were females. Data on race and ethnicity were available for 35% of the sample and, of this group, Black participants were overrepresented for both the low contact hour group (76.9%) and high contact hour group (87.9%). The mean BMI_z_ score was 2.43 for the low contact hour group and 2.41 for the high contact hour group. The mean BMI%_95_ for the high contact hour group was also lower than that of the low contact hour group (146.8 vs. 148.3). Eighty-six percent (*n* = 350) of the sample had severe obesity, and 13% had obesity (*n* = 54).

#### Distribution of Age in the Sample

The age distribution is presented in Table 2. The age of the participants ranged from 2–19 years, and 9-year-olds accounted for the highest prevalence (*n* = 55; 13.6%). Overall, the majority of the sample was in the 7–12 age range.

### 3.2. Bivariate Analyses

#### 3.2.1. Kaplan-Meir Analyses

**Figure 2:**
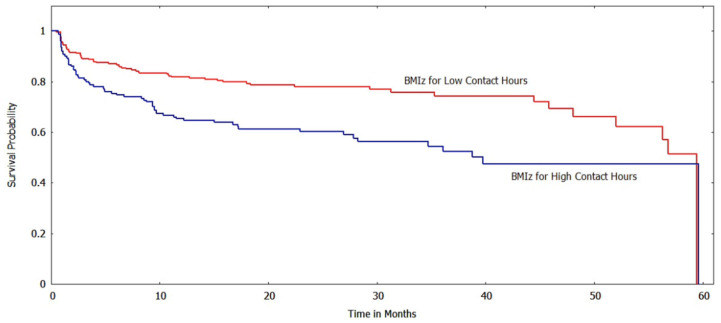
Survival curves for low vs. high contact hours patients with BMI_z_ as the risk factor (log-rank test: *p* < 0.001). The *y*-axis shows the survival probability (chance of experiencing an improved BMI state).

**Figure 3:**
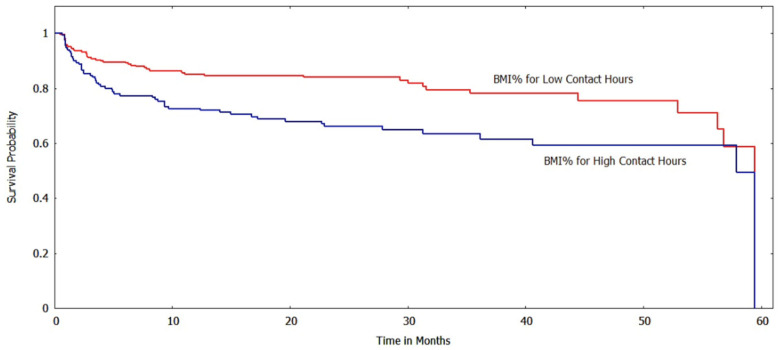
Survival curves for low vs. high contact hours patients with BMI%_95_ as the risk factor (log-rank test: *p* < 0.001). The *y*-axis shows the survival probability (chance of experiencing an improved BMI state).

**Figure 4:**
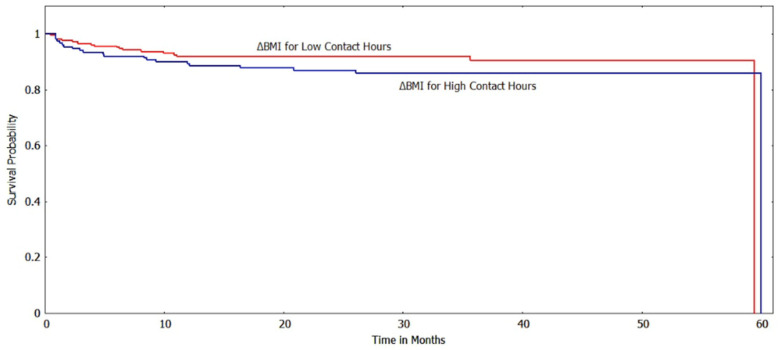
Survival curves for low vs. high contact hours patients with ΔBMI as the risk factor (log-rank test: *p* > 0.05). The *y*-axis shows the survival probability (chance of experiencing an improved BMI state).

The Kaplan-Meir method was used to estimate the survival function of the subgroups based on various risk factors/indicator endpoints. Figure 2 and Figure 3 show the survival curves of the low and high contact hour groups with improvements in BMI_z_ and BMI%_95_, respectively, as the outcome indicator. For both BMI metrics, the survival function indicates that the low contact hour group had greater survival probabilities (longer times to reach the event endpoint) than the high contact hour group, and a log-rank test found that the survival curves were significantly different (*p* < 0.001). When ΔBMI was used as a risk factor, unlike with the other BMI metrics, the survival curves of the low and high contact groups were not significantly different (*p* > 0.05) (Figure 4).

**Figure 5:**
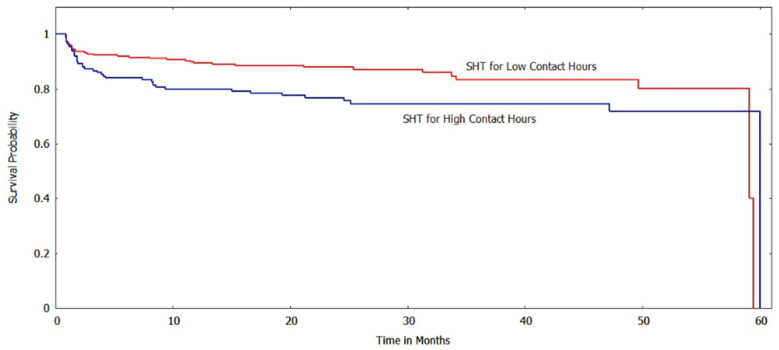
Survival curves for low vs. high contact hours patients with systolic hypertension (SHT) as the risk factor (log-rank test: *p* < 0.05). The *y*-axis shows the survival probability (chance of experiencing an improvement in systolic hypertension state).

**Figure 6:**
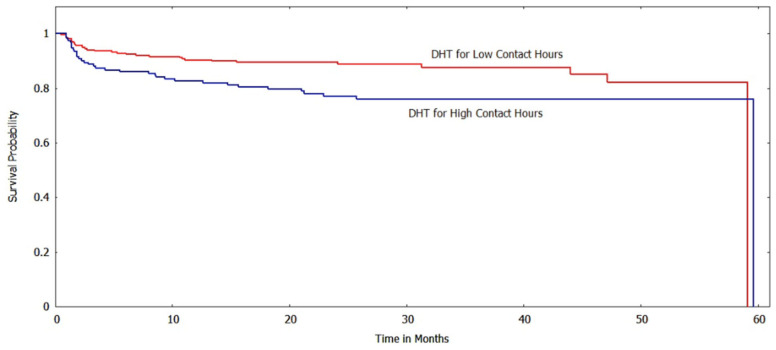
Survival curves for low vs. high contact hours patients with diastolic hypertension (DHT) as the risk factor (log-rank test: *p* < 0.01). The *y*-axis shows the survival probability (chance of experiencing an improvement in diastolic hypertension state).

Our study also used survival analysis to examine the association between contact hours and various cardiovascular and metabolic risk factors at the bivariate level. Using improvements in systolic hypertension for 12 consecutive months as an event endpoint, we found that the high contact hour group had lower survival probabilities than the low contact hour group. The survival curves were significantly different (*p* < 0.05). The survival function for diastolic hypertension based on the contact hour group also showed that the survival probabilities for the high contact hour group were lower, and a log-rank test found that the curves differed significantly (*p* < 0.01). (Figure 5 and Figure 6).

**Figure 7:**
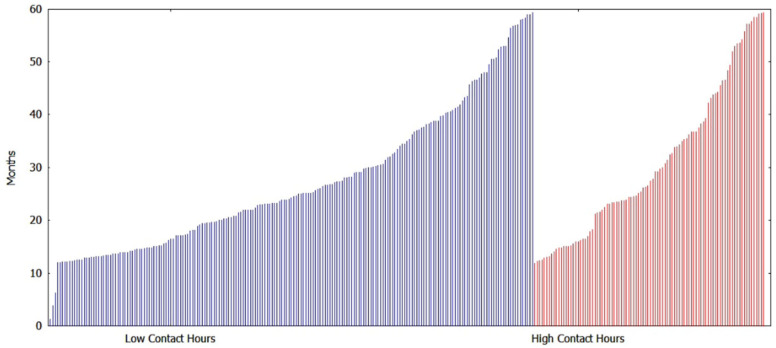
Censoring times of 201 low contact hour patients and 96 high contact hour patients on the last visit before the end of study at 60 months.

Figure 7 shows the distribution of censoring times of the patients on the last visit before the end of the study at 60 months (for BMI%_95_ risk factor) based on contact hour grouping. Overall, we see a greater number of censorings for children with low contact hours (201 patients) vs. high contact hours (96 patients).

#### 3.2.2. Mean Time Spent in the Improved State

We compared the mean time participants spent in the improved state (below the baseline for 12 months) for the subgroups by select risk factors. For all BMI risk factors studied, the low contact hour group spent a shorter time in the improved state. With BMI_z_ as a risk factor, the low contact hour group spent a mean time of 26.2 months, while the high contact hour group had a mean time of 34.8 months. This difference was statistically significant (*p* < 0.01) (Table 3).

#### 3.2.3. Mean Times Getting to the Event

We also noted the mean times participants took to reach the event. For both BMI_z_ and BMI%_95_, the high contact hour group took on average shorter times (8.45 vs. 11.2 months, and 8.88 vs. 11.2 months, for BMI_z_ and BMI%_95_, respectively) (Table 4).

#### 3.2.4. Multivariable Analyses

Cox PH regression was used to evaluate the independent effect of contact hours on the event endpoint (improvement in BMI for 12 consecutive months). We controlled for age as participants in the high contact hour group were significantly younger than those in the low contact hour group (Table 1). We also controlled for sex as prior studies have shown differences in obesity outcomes in children and adolescents based on sex [28,29].

Table 5 shows the models for BMI%_95_, BMI_z_ score, and ΔBMI. In all three BMI risk factor models, contact hours were independently associated with an increased hazard of improving BMI score for 12 consecutive months. The first model indicates that, when controlling for age and sex, participants exposed to high contact hours had over two times the “hazard” of achieving an improvement in BMI%_95_ score, compared to low contact hours participants (HR = 2.11, 95%CI (1.41–3.17), *p* < 0.001). According to this model, age was also an independent predictor of the event endpoint. Each additional year was associated with a 7% increase in the hazard of improving one’s BMI%_95_ score (HR = 1.07, 95%CI (1.001–1.13, *p* = 0.047).

While holding age and sex constant, there was an 81% increase in the hazard of improvement of BMI_z_ score for those in the high contact hour group, compared to participants in the low contact hour group (HR: 1.81, 95%CI (1.27–2.58), *p* = 0.001). The impact of age was reversed for BMI_z_. A one-year increase in age was associated with a 7% decrease in the hazard of improvement (HR: 0.929, 95%CI (0.875–0.987), *p* = 0.017) while holding sex and contact hours constant.

In the final model with ΔBMI as the outcome indicator, adjusting for sex and age, there was nearly a 2-fold increase in the hazard of improvement for high contact hour participants compared to those in the low contact hour group (HR: 1.92, 95%CI (1.03–3.60), *p* = 0.042). Sex was not a significant predictor of the event endpoint in any of the models.

## 4. Discussion

There is a paucity in the literature on long-term outcomes in childhood obesity in underserved patient populations Optimal strategies for evaluating long-term obesity interventions, as in the current study, have not been well-studied. To our knowledge, no published study has used the method of survival analysis to evaluate a long-term pediatric obesity intervention program. This statistical technique allowed us to model the hazard of improvement in BMI metrics at any given point. The survival analysis model incorporated data from subjects who experienced and did not experience the “event” (censored data) [22,23], and a key feature was that patients were able to enter at different times and followed through the end of the study.

Our study unfolded that contact hours were independently associated with the hazard of improving all three BMI metrics: BMI%_95_, BMI_z_, and ΔBMI. Of note, researchers have cautioned against the use of BMI_z_ scores as a metric for children with severe obesity [27,30,31]. Freedman et al. explained that different BMI values can map to the same z-score, which varies by sex and age. They further noted that BMI_z_ has weaker correlations to circumferences, skin fold, and fat mass, compared to BMI%_95_ or ΔBMI, in children with severe obesity [27].

In terms of the strength of the correlations of the various BMI measures, in the above study where Freedman et al. compared the relations of BMI_z_ and other BMI metrics to circumferences, skinfolds, and fat mass, the researchers found that BMI%_95_ was strongly correlated with modified BMI_z_ (r = 0.93) and ΔBMI (r = 0.98) [27]. On the other hand, weaker correlations were found for BMI_z_ with BMI%_95_ (r = 0.81) and ΔBMI (r = 0.87).

However, the current study used BMI_z_ scores as an indicator to evaluate to what extent the findings would deviate from those with BMI%_95_. Our results indicate that BMI_z_ scores may still be informative, and their use should be examined within the context of the specific study and the variables measured.

Age was also an independent predictor of the hazard of improvement of BMI%_95_ and BMI_z_. Our findings suggest that without the influence of contact hours, older participants are more likely to see improvements in BMI%_95_. We saw a reversal in the impact of the direction of age when BMI_z_ was used as the risk factor, indicating that younger children may have more success in improving their BMI_z_ scores.

Other researchers have investigated the effect of age in obesity intervention programs. Studies suggest that obesity behavioral treatment, applied in childhood, may be more successful than if introduced during adolescence [32,33]. Singer et al. analyzed the outcome of a 1-year lifestyle intervention program in 1291 children at the end of the intervention, and a year later, and have held that children with extreme obesity respond better to lifestyle interventions than extremely overweight adolescents [33]. These researchers found that children with extreme obesity ≤10 years showed a significantly greater reduction in BMI_z_ score (−0.24 ± 0.38), compared to extremely overweight adolescents (−0.16 ± 0.38), *p* = 0.021. Our findings suggest a parallel direction of the effect of age on the improvement of BMI_z_ scores.

One possible explanation for these findings is the role of parental involvement. Researchers explain that lifestyle interventions involving parents (typical with younger children) are more effective than interventions with limited parental involvement [33]. The implications of these findings could be applied to pediatric obesity programs in central Brooklyn, New York. Effectively addressing severe obesity in central Brooklyn may necessitate a robust early intervention, while actively incorporating parental involvement. Furthermore, a recent study has found that positive parental perceptions of weight were associated with improved compliance in the LLLR program [34]. These findings reiterate that parental involvement is a core component of pediatric lifestyle modification programs, which is an integral part of program success.

Some scholars have recommended the use of ΔBMI in lieu of BMI_z_ when studying severe childhood obesity. We found that like BMI%_95_ and BMI_z_, high contact hours were independently associated with the hazard of improvement of ΔBMI. However, age was not statistically significant when ΔBMI was used as a risk factor. These results suggest that flexibility may be required when determining what BMI metric is optimal for assessing severe obesity, as they can yield different results, in light of the particular study and the variables measured.

When we examined the average durations participants spent in the improved state, at the bivariate level, we found that the low contact hour group spent on average shorter times, compared to the high contact hour group. This finding is informative as it suggests that exposure to high contact hours is not only associated with a quicker attainment of the event endpoint, but also with the possible longevity and maintenance of that improved state.

This research has also unfolded that exposure to high contact hours was significantly associated with lower survival probabilities (shorter survival times) when improvements in systolic and diastolic hypertension were used as event endpoints. As shown in Table 1, patients presenting at LLLR have a range of abnormal cardiovascular and metabolic risk factors. Thus, the LLLR intervention model may be vital in addressing factors beyond BMI metrics.

A core strength of using survival analysis for this study is that it requires a shorter follow-up time to construct a robust sample for an evaluation analysis. Our previous evaluation study required a 14-year time frame (2002–2016) to construct a retrospective pre-post evaluation of 144 children [21]. In this current study, we are able to draw on more recent data in a shorter time span (2010–2016). As discussed earlier, we consider both loss-to-follow-up and end-of-study censoring. An important consideration is that even with considerable loss-to-follow-up censoring, the inclusion of the event times of the loss-to-follow-up subjects allowed enough study power to detect a significant positive association between greater contact hours and shorter times to event. In our previous study, we did not find a significant association between contact hours and metabolic outcomes [21].

Our findings on the efficacy of contact hours are consistent with that in the current literature. In the US Preventive Task Force’s review of 42 trials with 6956 participants, they found that comprehensive intensive behavioral interventions with ≥26 contact hours over a period of 2–12 months were associated with weight change. That review also found that ≥52 contact hours resulted in greater weight loss and improvements in cardiovascular and metabolic risk factors [35].

We have presented the outcome of the evaluation of an obesity intervention program in a hard-to-reach and low-adherent patient population in central Brooklyn, New York. Scholars have emphasized that obesity presents a complex problem, involving the broader social, physical, economic, and policy context [36]. Thus, to effectively address the obesity epidemic, resources and efforts should be geared towards these underserved areas to encourage patient compliance and substantial participation in disease management intervention programs.

### Limitations

This study is subject to several limitations. Most of the patients seen at LLLR are African American (85%) and Latino (14%), so these results may not be generalizable to all populations. However, these findings may be applied to youths with similar demographics in urban settings. Although age was statistically significant in the multivariate models, our findings on its effect should be interpreted in light of the low increased/reduced hazard of improving BMI metrics. Another limitation is that the study design does not account for recidivism. Further research should determine the relapse (if any) of study participants involved in similar disease management programs. This study is further limited by potential bias from confounding factors that were not controlled for (i.e., change in diet, self-efficacy, or increased physical activity at home or school). Future studies should incorporate this data in their assessment of an obesity management program. Additionally, future research should consider deconstructing obesity intervention programs to better understand the elements that drive program success or failure [37]. Despite these limitations, a major strength of this research is that it was a 5-year longitudinal study that utilized long-term data which is useful in evaluating the efficacy of an obesity treatment program. We assessed the three main BMI metrics in the current study for comparison. Our findings are informative for future research in that results may vary based on the variable measured, which must be considered when determining the optimal choice of BMI metric. Another strength of this study is that it provides vital data on real-world childhood obesity intervention in one of the most underserved locales in New York City, central Brooklyn. Childhood obesity research in these communities is lacking, and this study helps to grow the limited available literature.

## 5. Conclusions

This research has shown that for all measures of BMI: BMI%_95_, BMI_z_, or ΔBMI, contact hours have remained statistically significant in predicting the hazard of improvement. Exposure to high contact hours may also predict maintenance of the improved state (for BMI_z_), as well as improvement in systolic and diastolic hypertension. Given the immense burden of severe obesity, the AHA has called for novel treatment strategies for changing the health trajectories of youth with severe obesity [5]. Our findings indicate that chronic disease management such as childhood obesity may require a robust treatment model, similar to that employed at LLLR, which provides a structured treatment program that incorporates clinical care and lifestyle interventions while leveraging community resources. Furthermore, patients’ ongoing and substantial participation in a childhood obesity program is critical to their success. Significantly, targeted resources should be allocated to underserved communities like central Brooklyn to address childhood obesity and other health disparities.

## Figures and Tables

**Table 1:** Baseline characteristics and health indicators of the study population (*n* = 406).

	Low Contact Hours		High Contact Hours		
Variable	N (total)	Mean ± SD or N (%)	N (total)	Mean ± SD or N (%)	*p*-Value
Age in years, mean ± SD	256	11.0 ± 3.44	150	10.0 ± 2.66	**0.00**
Sex	256		150		
Male		111 (43.4%)		67 (44.7%)	0.84
Female		145 (56.6%)		83 (55.3%)	
Race	78		66		
Black		60 (76.9%)		58 (87.9%)	
Hispanic		15 (19.2%)		8 (12.1%)	
White		0 (0.00%)		0 (0.00%)	0.12
Asian		0 (0.00%)		0 (0.00%)	
Other/Unknown		3 (3.85%)		0 (0.00%)	
BMIz, mean ± SD	256	2.43 ± 0.44	150	2.41 ± 0.42	0.72
BMI%_95,_ mean ± SD	256	148.3 ± 28.4	150	146.8 ± 26.4	0.60
Obesity Prevalence	256		150		
Healthy weight		0 (0.00%)		1 (0.66%)	
Overweight		0 (0.00%)		1 (0.66%)	0.19
Obese		38 (14.8%)		16 (10.7%)	
Severely obese		218 (85.2%)		132 (88.0%)	
Abnormal Waist circumference	210	195 (92.9%)	133	124 (93.2%)	0.89
Hypertension					
Systolic Hypertension	237	70 (29.5%)	145	48 (33.1%)	0.49
Diastolic Hypertension	237	40 (16.9%)	144	19 (13.2%)	0.38
Lipid Levels					
Abnormal Total cholesterol	195	75 (38.5%)	129	50 (38.8%)	0.96
Abnormal High-density lipoprotein	189	65 (34.4%)	122	34 (27.9%)	0.23
Abnormal Low-density lipoprotein	185	56 (30.3%)	121	33 (27.3%)	0.57
Abnormal Triglycerides	188	62 (33.0%)	120	30 (25.0%)	0.14
Elevated fasting glucose	172	18 (10.5%)	114	8 (7.02%)	0.32
Presence of 3 or more components of the Metabolic syndrome	135	36 (26.0%)	96	24 (25.0%)	0.78

Bold means statistically significant at the 5% level

**Table 2:** Age distribution at baseline (*n* = 406).

Age	Frequency	Percent
2	1	0.25
3	2	0.49
4	9	2.22
5	12	2.96
6	21	5.17
7	42	10.3
8	39	9.61
9	55	13.6
10	47	11.6
11	50	12.3
12	37	9.11
13	27	6.65
14	19	4.68
15	20	4.93
16	15	3.69
17	4	0.99
18	2	0.49
19	4	0.99

**Table 3:** Total number of people that experienced the “event” and mean times (in months) of staying in the improved state for the BMI metrics based on contact hour group grouping.

Risk Factor	Contact Hour Group	N	Mean	Std. Dev.	Min	Max	*p*-Value
BMI_z_	Low	61	26.2	15.3	12.7	103.6	0.00
	High	65	34.8	17.1	12.2	79.3	
	(Total)	126	30.6	16.7	12.2	103.6	
BMI%_95_	Low	48	28.6	17.3	12.7	103.6	0.43
	High	54	31.2	15.7	12.2	85.5	
	(Total)	102	30.0	16.5	12.2	103.6	
ΔBMI	Low	21	23.3	9.25	12.2	43.8	0.47
	High	21	21.4	7.43	12.8	44.7	
	(Total)	42	22.4	8.34	12.2	44.7	

**Table 4:** Total number of people that experienced the “event” and mean times (in months) getting to the event for the BMI risk factors based on contact hour grouping.

Risk Factor	Contact Hour Group	N	Mean	Std. Dev.	Min	Max	*p*-Value
BMIz	Low	61	11.2	15.3	0.47	56.8	0.24
	High	65	8.45	10.1	0.67	39.8	
	(Total)	126	9.78	12.9	0.47	56.8	
BMI%_95_	Low	48	11.2	15.7	0.47	56.8	0.39
	High	54	8.88	11.7	0.07	57.9	
	(Total)	102	10.0	13.7	0.47	57.9	
ΔBMI	Low	21	6.41	7.64	0.47	35.6	0.88
	High	21	6.74	7.17	0.93	26.0	
	(Total)	42	6.58	7.32	0.47	35.6	

**Table 5:** Cox PH Regression for contact hours predicting improvement in BMI (*n* = 406).

	BMI%_95_		BMIz		ΔBMI	
Variable	HR (95% CI)	*p*-value	HR (95% CI)	*p*-value	HR (95% CI)	*p*-value
Contact Hour Group (Referent—Low)	2.11 (1.41–3.17)	<0.001	1.81 (1.27–2.58)	0.001	1.92 (1.03–3.60)	0.042
Age	1.07 (1.001–1.13)	0.047	0.929 (0.875–0.987)	0.017	1.10 (0.994–1.21)	0.07
Sex (Referent—Male)	0.929 (0.625–1.38)	0.714	1.31 (0.908–1.88)	0.149	1.26 (0.676–2.35)	0.47

## Data Availability

Data is unavailable. Confidential clinical data were used for this study.
